# Characterization of recombinant IgA producing CHO cell lines by qPCR

**DOI:** 10.1186/1753-6561-7-S6-P114

**Published:** 2013-12-04

**Authors:** David Reinhart, Wolfgang Sommeregger, Monika Debreczeny, Elisabeth Gludovacz, Renate Kunert

**Affiliations:** 1 Vienna Institute of BioTechnology, Department of Biotechnology, University of Natural Resources and Life Sciences, Muthgasse 11, 1190 Vienna, Austria; 2 Vienna Institute of BioTechnology, Imaging Center, University of Natural Resources and Life Sciences, Muthgasse 11, 1190 Vienna, Austria

## Abstract

Immunoglobulin A (IgA) mediates a key role in mucosal immunity and is a promising novel immunotherapeutic candidate. However, difficulties in obtaining enough material often hamper *in vivo *explorations. We have previously generated recombinant Chinese hamster ovary (CHO) cell lines which expressed two different HIV-1 antibodies, 3D6 and 4B3, as IgA1 [1]. One cell line (3D6-IgA) shows high production rates, whereas the other (4B3-IgA) secretes rather low amounts of product. In order to unravel the mystery of productivity bottlenecks we extensively characterized the cell lines regarding growth rate, IgA productivity in long-term culture, immunofluorescence microscopy, flow cytometry and Western blotting of intra- and extracellular product (data not shown). The generated data encouraged us to analyze whether the observed antibody productivities could be explained by gene copy number (GCN) or mRNA levels.

## Materials and methods

CHO host (ATCC CRL-9096) and recombinant cell lines [1] were cultivated in spinner vessels (Techne, UK) with 50 mL medium (ProCHO5, Switzerland), at 37°C and 50 rpm.

Genomic DNA (gDNA) was isolated from 2 × 10^6 ^cells using the DNA Blood Mini Kit (Qiagen, Netherlands) according to the manufacturers' instructions. Quantification was performed spectrophotometrically at an absorbance of 260 nm and the purity was determined by measuring the ratio at 260 nm and 280 nm. gDNA samples were stored at 4°C. Cellular RNA was isolated from 5 × 10^6 ^cells using the Ambion Tri Reagent Solution (Life Technologies, CA) according to the manufacturers' instructions. To remove DNA contaminations from extracted RNA the preparation was digested with 3 U DNase I (Qiagen, Netherlands) for 30 min at RT together with 160 U RNase inhibitor (Life Technologies, CA) and then inactivated for 10 min at 75°C before another RNA precipitation step. Purified total RNA was dissolved in 25 μl RNase free water containing 60 U RNase inhibitor. cDNA was obtained by reverse transcription. 1.5 μg RNA, 1 μg random primers (Promega, WI) and 12.5 nmol dNTPs (New England Biolabs, MA) were incubated in a reaction volume of 14 μl for 5 min at 70°C and 2 min at room temperature. Then, 40 U RNase inhibitor, 200 U M-MLV reverse transcriptase and buffer (both Promega, WI) were added to a reaction volume of 20 μl and incubated for 30 min at 37°C before denaturation for 5 min at 95°C.

Real-time PCR (qPCR) analysis was performed on a MiniOpticon qPCR device (Biorad, CA). Primers and the fluorogenic hydrolysis probes were synthesized by Sigma (MO). Same primers and probes were used for the analysis of gDNA and cDNA. The reaction mix included iQ Supermix (Biorad, CA), 6 pmol primer and 4 pmol hydrolysis probe for HC, JC and ß-actin quantification or 12 pmol primer and 8 pmol hydrolysis probe for LC determination in 20 μl reaction volume. 3 ng pre-denatured (99°C, 10 min) gDNA or 3 μL cDNA from a 1:50 dilution of the reverse transcription reaction was used directly for qPCR. Negative controls (NC), no template controls (NTC) and no reverse transcriptase controls (NRT) for transcript analysis were included in each run. The quantification cycle (Cq) was determined by linear regression and baseline subtraction using the CFX Manager (Biorad, CA). The mean qPCR efficiencies for HC, LC, JC and ß-actin were calculated from raw fluorescence data using the LinRegPCR software application, V12.17 [2]. Quantification was done by relative quantification with efficiency correction [3] using ß-actin as internal reference and expressed as ratios.

## Results and discussion

**Figure 1:**
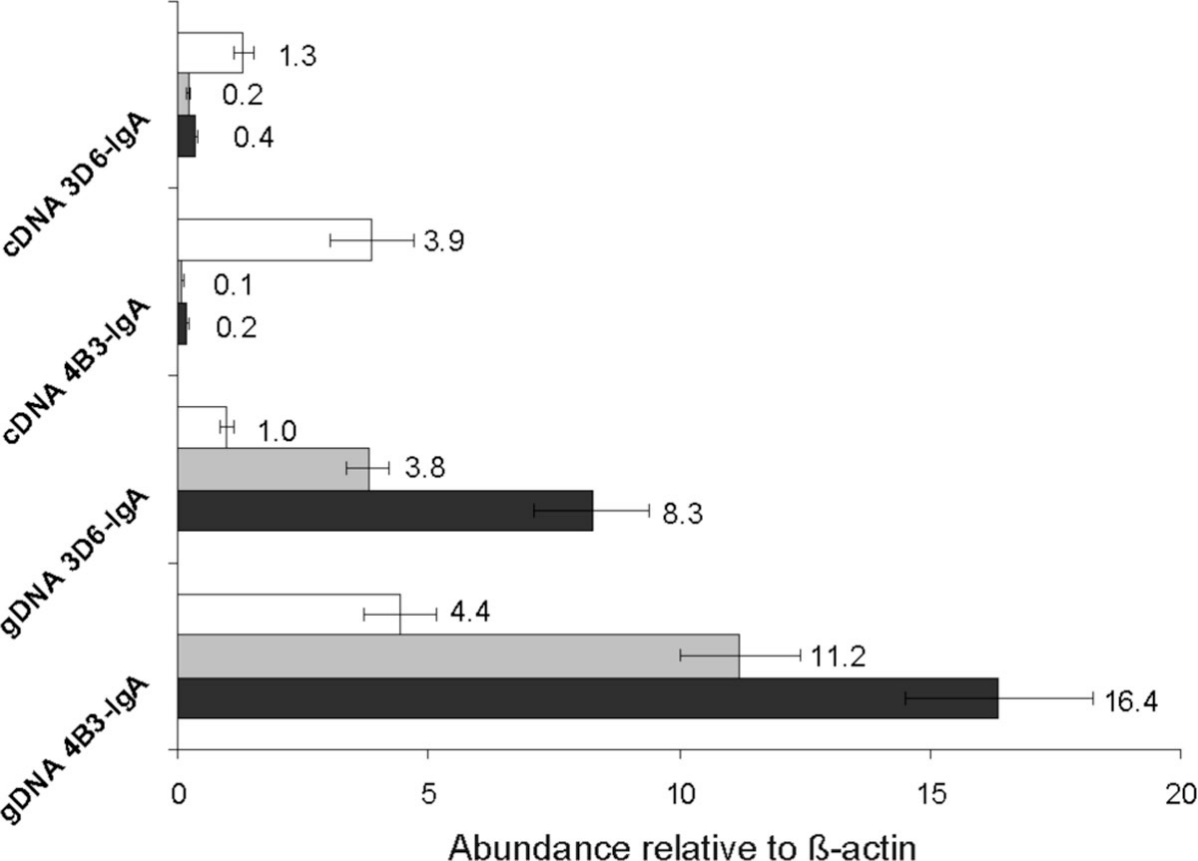
**Gene copy number and transcript level of recombinant clones expressing 3D6-IgA or 4B3-IgA**. The abundance of LC (), JC () and HC () genes was calculated relative to ß-actin.

qPCR was performed in six technical replicates. The Cq values and calculated efficiencies were well reproducible (Table 1). gDNA analysis revealed an overall higher exogenic GCN for the low producer 4B3-IgA than for 3D6-IgA (Figure 1). On the genomic level clone 4B3-IgA contained two times more HC, three times more JC and four times more LC than 3D6-IgA. Both clones incorporated more HC genes than JC than LC. This could be due to the presence of the dhfr amplification gene on the HC plasmid, whereas the neomycin resistance gene was located on the JC plasmid. No selection marker was included on the LC plasmid.

**Table 1:** Calculated efficiencies (E), Cq and ΔCq values and copies relative to ß-actin for gDNA and cDNA derived from clones 3D6-IgA and 4B3-IgA

GOI	Target	Clone	Cq	max. SD [%]	E	SD (%)	ΔCq ß-actin	Copies relative to ß-actin
ß-actin	gDNA	3D6-IgA	24.60	0.20	2.07	2.22	n/a	n/a
		4B3-IgA	24.21	0.14	2.07	2.22	n/a	n/a
	cDNA	3D6-IgA	18.52	0.13	2.03	0.43	n/a	n/a
		4B3-IgA	16.25	0.63	2.04	1.33	n/a	n/a

HC	gDNA	3D6-IgA	23.56	0.16	1.95	3.32	-1.03	8.28
		4B3-IgA	22.11	0.14	1.95	3.32	-2.11	16.44
	cDNA	3D6-IgA	21.78	0.17	1.91	1.35	3.26	0.38
		4B3-IgA	19.50	0.68	1.97	1.53	3.25	0.20

JC	gDNA	3D6-IgA	24.81	0.03	1.95	0.94	0.22	3.80
		4B3-IgA	22.77	0.10	1.95	0.94	-1.44	11.20
	cDNA	3D6-IgA	24.52	0.23	1.82	0.87	5.97	0.22
		4B3-IgA	20.81	1.54	1.96	0.27	4.56	0.10

LC	gDNA	3D6-IgA	24.90	0.14	2.05	0.59	0.31	0.98
		4B3-IgA	21.50	0.21	2.11	1.21	-2.71	4.40
	cDNA	3D6-IgA	20.26	0.20	1.88	0.75	1.73	1.30
		4B3-IgA	15.02	2.36	1.98	1.30	-1.22	3.93

mRNA levels were additionally quantified by qPCR to exclude any misinterpretation of our analysis due to incompletely transfected expression cassettes, chromosomal position effects or transgene silencing. Despite higher gene copy numbers 4B3-IgA contained only half of HC and JC transcripts as compared to 3D6-IgA. LC was transcribed with the same range of efficiency and resulted in three times more LC mRNA copies. In contrast to gDNA results, LC mRNA content greatly exceeded that of HC and JC in both clones (Figure 1). Hence, LC content, which has been proposed to be critical for high antibody productivities [4], should not have been limited by mRNA. Summarized, the respective mRNA levels differed slightly between the two recombinant cell lines, but were presumably not sufficient for the low specific productivity of clone 4B3-IgA.

## Conclusions

An overall higher exogenic GCN was determined for the low producer 4B3-IgA as compared to 3D6-IgA. Both clones incorporated more HC genes than JC than LC. Despite higher GCNs 4B3-IgA contained only half of HC and JC mRNA transcripts as compared to 3D6-IgA. LC was transcribed with similar efficiencies whereas LC mRNA content greatly exceeded that of HC and JC in both clones. All in all, differences in specific productivity, intracellular antibody chain content and volumetric titers of the cell lines could not sufficiently be explained by qPCR data of GCN and mRNA levels. Therefore, bottlenecks are believed to occur further upstream in the translational and/or protein processing machinery.
